# Effects of roxadustat on anemia, iron metabolism, and lipid metabolism in patients with non-dialysis chronic kidney disease

**DOI:** 10.3389/fmed.2023.1071342

**Published:** 2023-02-22

**Authors:** Keiji Hirai, Shohei Kaneko, Saori Minato, Katsunori Yanai, Momoko Hirata, Taisuke Kitano, Kiyonori Ito, Yuichiro Ueda, Susumu Ookawara, Yoshiyuki Morishita

**Affiliations:** Division of Nephrology, First Department of Integrated Medicine, Saitama Medical Center, Jichi Medical University, Saitama, Japan

**Keywords:** roxadustat, anemia, cholesterol, triglyceride, chronic kidney disease, erythropoiesis-stimulating agent

## Abstract

**Background:**

We determined the effects of roxadustat on the values of anemia, iron metabolism, renal function, proteinuria, and lipid metabolism and identified the associated factors of the change in hemoglobin levels after roxadustat administration in non-dialysis chronic kidney disease (CKD) patients who were receiving an erythropoietin-stimulating agent (ESA).

**Methods:**

We conducted retrospective analysis of the changes in hemoglobin, serum ferritin, total cholesterol (TC), low-density lipoprotein cholesterol (LDL-C), high-density lipoprotein cholesterol (HDL-C), and triglyceride levels; transferrin saturation; the estimated glomerular filtration rate; and the urinary protein/creatinine ratio over 24 weeks after the change from an ESA to roxadustat in 50 patients with non-dialysis CKD and anemia (roxadustat group). Seventy-two patients with non-dialysis CKD and anemia who proceeded ESA therapy were used as the control (ESA) group.

**Results:**

We observed no significant between-group differences in clinical parameters at baseline except for the significantly lower hemoglobin concentration and lower proportion of diabetes mellitus in the roxadustat group. The hemoglobin concentration was significantly higher in the roxadustat group after 24 weeks (11.3 ± 1.2 versus 10.3 ± 1.0 g/dL; *value of p* < 0.05), whereas the transferrin saturation, ferritin concentration, estimated glomerular filtration rate, and urinary protein/creatinine ratio were not different between the two groups. TC (135.9 ± 40.0 versus 165.3 ± 38.4 mg/dL; *value of p* < 0.05), LDL-C (69.1 ± 28.3 versus 87.2 ± 31.5 mg/dL; *value of p* < 0.05), HDL-C (41.4 ± 13.5 versus 47.2 ± 15.3 mg/dL; *value of p* < 0.05), and triglyceride concentrations (101.5 ± 52.7 versus 141.6 ± 91.4 mg/dL, *value of p* < 0.05) were significantly lower in the roxadustat group compared with the ESA group at 24 weeks. Multiple linear regression analysis showed that the roxadustat dose at baseline (standard coefficient [β] = 0.280, *value of p* = 0.043) was correlated with the change in the hemoglobin levels during the first 4 weeks of roxadustat treatment, whereas age (β = 0.319, *value of p* = 0.017) and the roxadustat dose at 24 weeks (β = −0.347, *value of p* = 0.010) were correlated with the hemoglobin concentration after 24 weeks of roxadustat administration.

**Conclusion:**

Roxadustat can improve anemia and reduce serum cholesterol and triglyceride levels in non-dialysis CKD patients after the patients’ treatment was switched from an ESA without affecting renal function or proteinuria. These results indicate that roxadustat has superior effects to ESAs regarding anemia and lipid metabolism at the dose selected for the comparison in patients with non-dialysis CKD.

## Introduction

Anemia is a frequently observed coexisting disease in patients with non-dialysis chronic kidney disease (CKD), and it is associated with faster decline in renal function, worse quality of life, and greater risk of mortality (1–3). Therefore, optimal and effective treatments of anemia are desired for maintaining renal function and improving the value of life and the prognosis of patients with non-dialysis CKD.

Erythropoietin-stimulating agents (ESAs) have been widely and effectively used to sustain optimal hemoglobin levels in patients with non-dialysis CKD (4). However, ESAs are administered subcutaneously, resulting in the patient’s pain.

Roxadustat is a novel and oral hypoxia-inducible factor (HIF) prolyl hydroxylase inhibitor which raises endogenous erythropoietin concentrations by stabilizing HIF (5), and it has been recently endorsed for the treatment of anemia in patients with non-dialysis CKD (6). A phase 3 clinical trial revealed that roxadustat raised the hemoglobin concentration after patients with non-dialysis CKD were switched from an ESA (7). However, there are few data regarding roxadustat’s effects on renal function and proteinuria. It is also not known which factors are associated with the change in hemoglobin levels after roxadustat treatment is initiated. Another phase 3 clinical trial reported that roxadustat reduced cholesterol and triglyceride concentrations in patients on hemodialysis (8). However, the effect of roxadustat on lipid metabolism has not been fully investigated in patients with non-dialysis CKD. Therefore, we conducted the present study to investigate roxadustat’s effects on renal function, proteinuria, and lipid metabolism and determined the associated factors of the change in hemoglobin levels after roxadustat administration, in addition to roxadustat’s effects on anemia and iron metabolism in non-dialysis patients who were receiving ESA on a clinical practice.

## Methods

### Ethical approval

Due to the retrospective nature of this study, the requirement for patients’ informed consent was not applicable. Information about the study was posted on display boards in our institution’s patient reception rooms informing patients of their right to withdraw from the study. The Ethics Committee of Saitama Medical Center, Jichi Medical University approved the study (RIN 22–004), which was performed in accord with the Declaration of Helsinki.

### Participants

The data of the patients who had been medicated in 2020–2021 at Saitama Medical Center, Jichi Medical University were collected. The study’s inclusion criteria were: (i) age more than 20 years, (ii) estimated glomerular filtration rate (eGFR) less than 60 mL/min/1.73 m^2^ (i.e., CKD stage G3 to G5), and (iii) having been treated with an ESA for at least 48 weeks or with roxadustat for at least 24 weeks after being treated with an ESA for at least 24 weeks. The following exclusion criteria were applied; patients who were undergoing or who had undergone hemodialysis, peritoneal dialysis, renal transplantation, malignancy, or red blood cell transfusion, and those who had shown poor compliance with their roxadustat treatment.

### Study design

**Figure 1:**
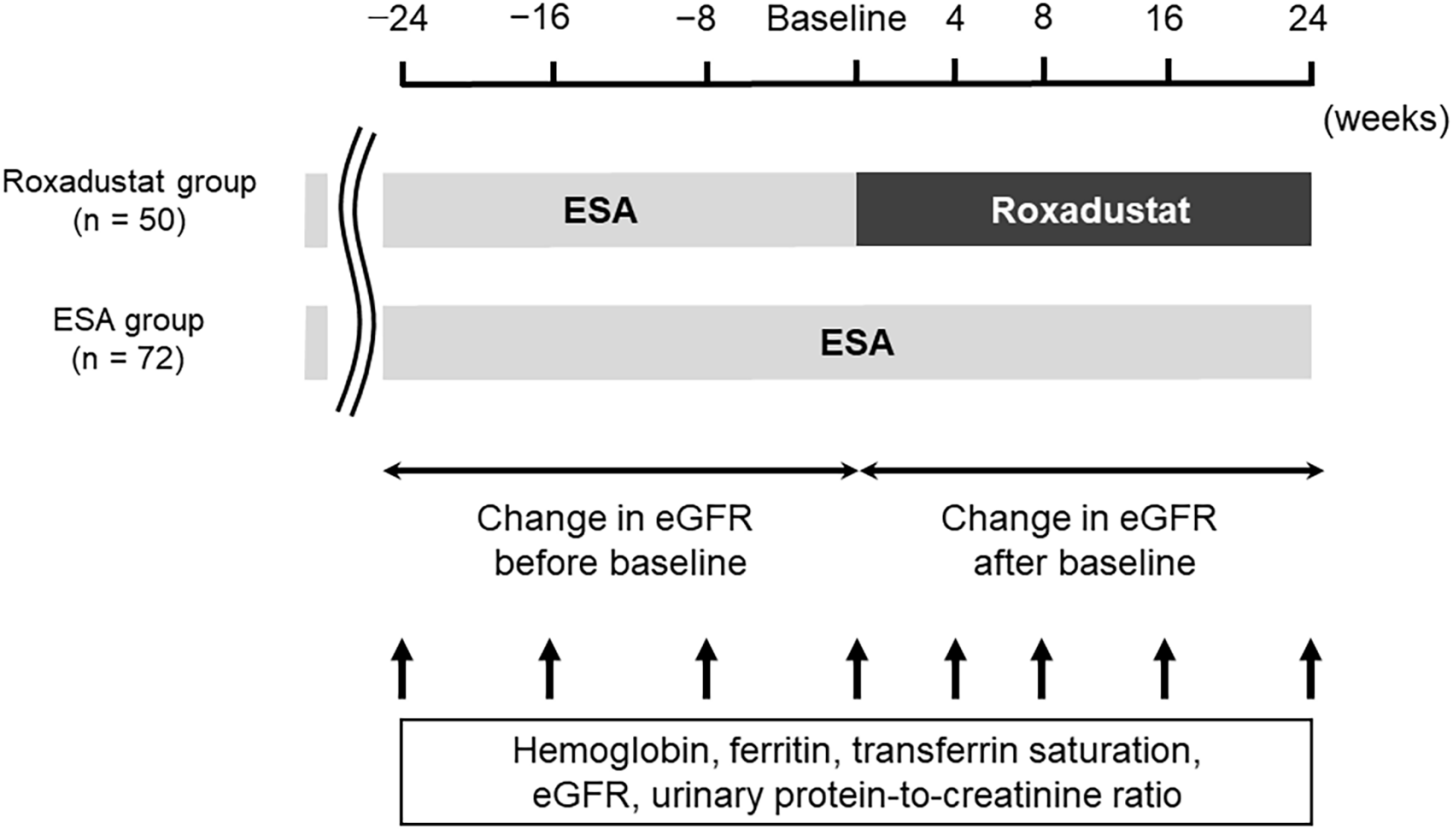
Study design. eGFR, estimated glomerular filtration rate; ESA, erythropoiesis-stimulating agent.

A total of 122 patients were enrolled in this retrospective comparative study. Figure 1 illustrates the study design. Each patient’s demographical and clinical data were gained from his or her medical charts. Fifty patients who had been treated with roxadustat for at least 24 weeks after being treated with an ESA for at least 24 weeks were assigned to the roxadustat group. The 72 patients who had been treated with an ESA for at least 48 weeks were assigned to the ESA group (control group). The date that was considered the baseline for each of the ESA-group patients was between December 1, 2020 to March 1, 2021, during which the patients of roxadustat-group started receiving roxadustat. Roxadustat was administered orally, and the roxadustat-group patients took it 3×/week. at bedtime. The ESAs were administered subcutaneously 1×/month or 1×/2 months on the day of a hospital visit. In both the roxadustat and ESA groups, we evaluated the changes in hemoglobin, serum ferritin, total cholesterol (TC), low-density lipoprotein cholesterol (LDL-C), high-density lipoprotein cholesterol (HDL-C), triglyceride concentrations, transferrin saturation (TSAT), eGFR, and urinary protein/creatinine ratio from baseline to 24 weeks later. We conducted a multiple linear regression analysis to identify associated factors of the change in the hemoglobin levels during the first 4 weeks of roxadustat treatment and the hemoglobin concentration after 24 weeks of roxadustat treatment.

### Laboratory methods

The Saitama Medical Center’s clinical laboratory measured the patients’ urine and blood parameters. The erythropoietin resistance index (ERI) was determined as the mean weekly dosage of epoetin (IU)/body mass (kg)/hemoglobin (g/dL) (9). For the conversion of ESA doses to epoetin doses, ratios of 225:1 and 200:1 were used for epoetin beta pegol and darbepoetin alfa, respectively (10).

### Statistics

The data of the continuous variables were shown as the mean plus/minus standard deviation when they were normally distributing; those of the not normally distributing continuous variables were shown as the median and interquartile range. The data of the categorical variables were presented as numbers and percentages. We compared the roxadustat and control groups’ clinical data by performing Student’s *t*-test (for normally distributing data) and the Mann–Whitney *U*-test (for not normally distributing data). Fisher’s exact test was used to compare the groups’ component ratios. A repeated-measures analysis of variance accompanied by Tukey’s test was conducted to compare the serial measurements within each of the two groups. We performed a linear regression analysis to determine the change rate in the patients’ eGFR and calculated it as the slope (monthly) for each patient before the baseline and after the baseline. We used the paired t-test to compare the eGFR change rate before and after the baseline in the roxadustat and ESA groups. In a multiple linear regression analysis, we included the parameters that were significantly correlated with the change in hemoglobin levels during the first 4 weeks of roxadustat treatment and the hemoglobin concentration after 24 weeks of roxadustat treatment, in order to identify the variables that were independently correlated with the change in hemoglobin levels during the first 4 weeks of roxadustat treatment and the hemoglobin concentration after 24 weeks of roxadustat treatment. Probability (*p*)-values <0.05 were accepted as significant. All of the statistical analyses were conducted using JMP ver. 11 (SAS, Cary, NC, United States).

## Results

### Patient characteristics

**Figure 2:**
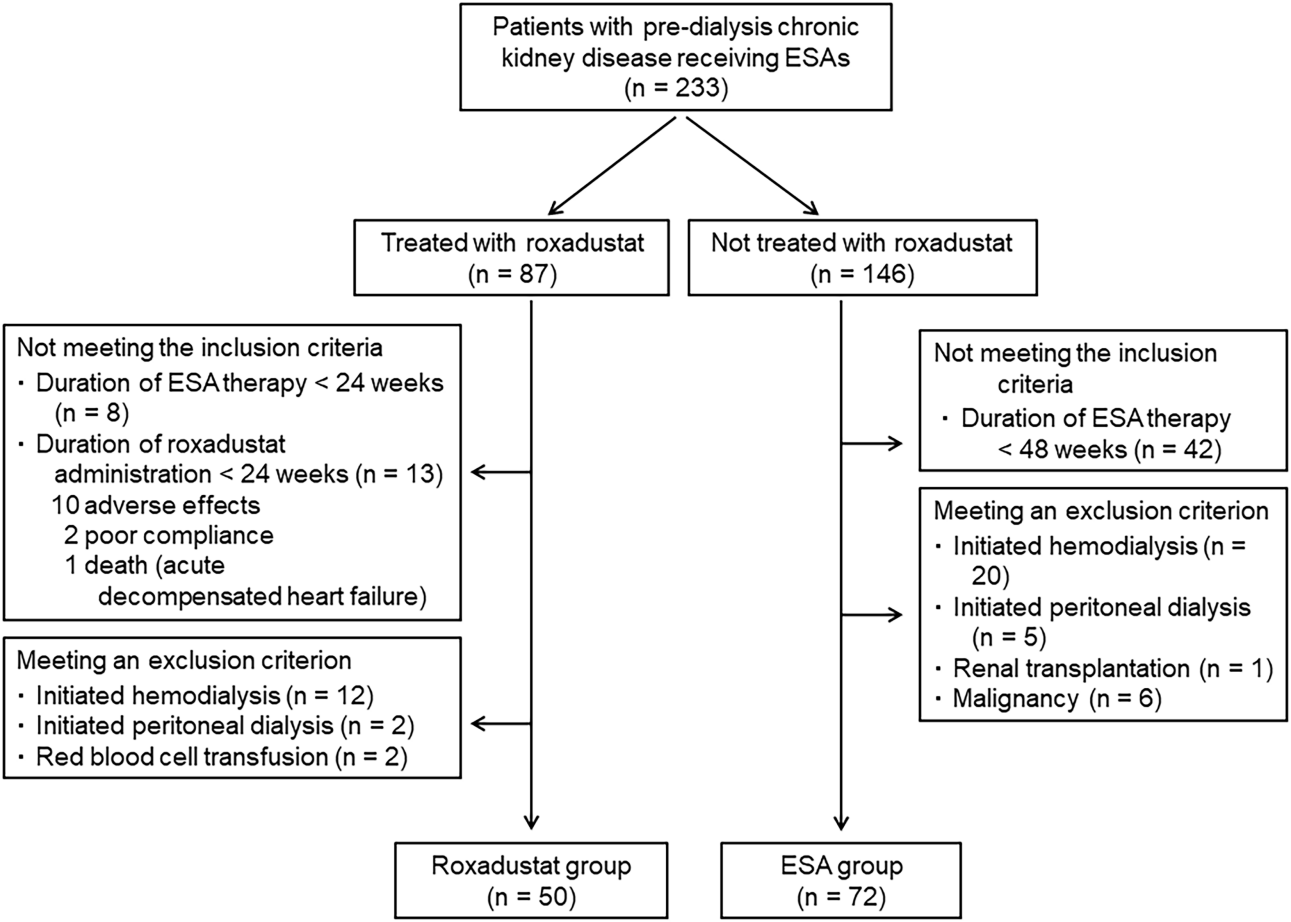
Patient flow diagram. ESA, erythropoiesis-stimulating agent.

In total, 233 patients with non-dialysis CKD who were receiving an ESA were identified. Of these 233 patients, 87 were treated with roxadustat after being treated with an ESA, and 146 were treated with only an ESA. Twenty-one of the patients treated with roxadustat did not fulfill the criteria of inclusion. Sixteen patients met at least one of the criteria of exclusion. The roxadustat group was thus comprised of 50 patients. The reasons for switching an ESA to roxadustat were resistance to ESA treatment in 9 patients and injection pain in 41 patients. Among the ESA-alone patients, 42 did not fulfill the criteria of inclusion, and 32 patients met at least one of the criteria of exclusion; the ESA group was thus the remaining 72 patients (Figure 2). Therefore, we analyzed the data of 122 patients (72 men and 50 women, mean age = 74.0 ± 11.3 years, mean body mass index = 23.9 ± 4.4 kg/m^2^). The mean baseline eGFR was 16.1 ± 8.8 mL/min/1.73 m^2^, and their CKD stages were as follows: stage G3a, 2 (1.6%) patients; stage G3b, 6 (4.9%) patients; stage G4, 50 (41.0%) patients; and stage G5, 64 (52.5%) patients. The mean values of hemoglobin and ferritin concentrations and TSAT at baseline were 10.2 ± 1.0 g/dL, 125.6 ± 115.1 ng/mL, and 32.7 ± 12.2%, respectively. Sixty-seven patients had received darbepoetin alfa and 55 had received epoetin beta pegol every 1 or 2 months. The mean ESA dose was 2,852 ± 2,343 IU/wk., and the mean ERI was 4.9 ± 4.2. A history of diabetes mellitus was present in 45.1%, myocardial infarction in 18.9%, and stroke in 7.4% of the participants. The percentages of participants receiving each medicine were as follows: calcium-containing (based) phosphate binder, 10.7%; calcium-free phosphate binder, 5.7%; vitamin D analog, 21.3%; iron supplement, 15.6%; zinc supplement, 12.3%; statin, 56.6%; and ezetimibe, 5.7%. Iron supplementation was newly started after roxadustat initiation in 10 patients in the roxadustat group, but iron supplementation was not changed in the ESA group because iron stores were sufficient (ferritin level ≥ 100 μg/l or TSAT ≥20%) (4). Table 1 summarizes the patient characteristics and medications at baseline in both groups; only the proportion of patients with diabetes mellitus and the hemoglobin concentration differed significantly between the roxadustat and ESA groups.

**Table 1:** Baseline patient characteristics.

		Roxadustat group (*n* = 50)	ESA group (*n* = 72)	*p* value
Age (years)		75.6 ± 9.4	72.8 ± 12.4	0.24
Male sex, n (%)		32 (64.0)	40 (55.6)	0.45
Body mass index (kg/m^2^)		23.2 ± 3.9	24.4 ± 4.6	0.25
Systolic blood pressure (mmHg)		142.8 ± 18.7	137.2 ± 17.2	0.16
Diastolic blood pressure (mmHg)		74.1 ± 12.6	74.1 ± 15.6	0.85
Diabetes mellitus, n (%)		17 (34.0)	38 (52.8)	0.044*
Hypertension, n (%)		46 (92.0)	61 (84.7)	0.27
Previous myocardial infarction, n (%)		7 (14.0)	16 (22.2)	0.35
Previous stroke, n (%)		4 (8.0)	5 (6.9)	1.00
Calcium-containing phosphate binder, n (%)		4 (8.0)	9 (12.5)	0.56
Calcium-free phosphate binder, n (%)		4 (8.0)	3 (4.2)	0.44
Vitamin D analog, n (%)		8 (16.0)	18 (25.0)	0.27
Iron supplement, n (%)		6 (12.0)	13 (18.1)	0.45
Zinc supplement, n (%)		6 (12.0)	9 (12.5)	1.00
Statin, n (%)		28 (56.0)	41 (56.9)	1.00
Ezetimibe, n (%)		3 (6.0)	4 (5.6)	1.00
Serum creatinine (mg/dL)		3.8 ± 1.6	3.5 ± 1.9	0.25
Estimated glomerular filtration rate (mL/min/1.73m^2^)		14.4 ± 6.7	17.2 ± 9.9	0.17
Chronic kidney disease stage, n (%)	G3a	0 (0.0)	2 (2.8)	0.64
	G3b	2 (4.0)	4 (5.6)	
	G4	19 (38.0)	31 (43.1)	
	G5	29 (58.0)	35 (48.6)	
Urinary protein excretion (g/gCr)		2.2 [0.8–3.7]	1.6 [0.6–3.3]	0.22
Hemoglobin A1c (%)		5.9 ± 0.8	6.2 ± 0.9	0.15
Total cholesterol (mg/dL)		170.5 ± 32.4	169.1 ± 37.8	0.65
Low-density lipoprotein-cholesterol (mg/dL)		88.1 ± 22.6	89.4 ± 30.7	0.86
High-density lipoprotein-cholesterol (mg/dL)		50.5 ± 17.0	49.7 ± 15.6	0.87
Triglyceride (mg/dL)		132.2 ± 78.9	137.1 ± 114.9	0.82
Uric acid (mg/dL)		6.0 ± 1.5	6.4 ± 1.6	0.14
Albumin (g/dL)		3.9 ± 0.4	3.8 ± 0.4	0.27
Hemoglobin (g/dL)		9.8 ± 1.0	10.4 ± 1.0	<0.001*
Sodium (mEq/L)		140.0 ± 2.7	139.3 ± 3.4	0.30
Potassium (mEq/L)		5.0 ± 0.5	4.8 ± 0.6	0.16
Chloride (mEq/L)		108.3 ± 3.3	107.1 ± 3.8	0.19
Total calcium (mg/dL)		8.7 ± 0.6	8.6 ± 0.5	0.41
Phosphorus (mg/dL)		4.3 ± 0.8	4.3 ± 1.1	0.63
Magnesium (mg/dL)		2.1 ± 0.4	2.1 ± 0.4	0.71
Brain natriuretic peptide (pg/mL)		98.8 [33.7–157.2]	58.2 [37.7–205.1]	0.96
C-reactive protein (mg/dL)		0.09 [0.06–0.16]	0.09 [0.05–0.17]	0.66
Ferritin (ng/mL)		94.9 [54.0–161.9]	92.4 [64.6–140.0]	0.95
Transferrin saturation (%)		33.0 ± 14.8	32.5 ± 10.3	0.65
ESA, n (%)	Darbepoetin alfa	29 (58.0)	38 (52.8)	0.58
	Epoetin beta pegol	21 (42.0)	34 (47.2)	
ESA dose (IU/week)		2000 [1000–3,750]	2,500 [1500–5,000]	0.35
Erythropoietin resistance index (IU/week/kg/(g/dL))		4.6 ± 4.3	4.9 ± 4.2	0.48

ESA, erythropoiesis-stimulating agent; IU, international units. Data are presented as the mean ± standard deviation, median [interquartile range], or number (%). *Statistically significant.

### Changes in ESA and roxadustat doses

**Figure 3:**
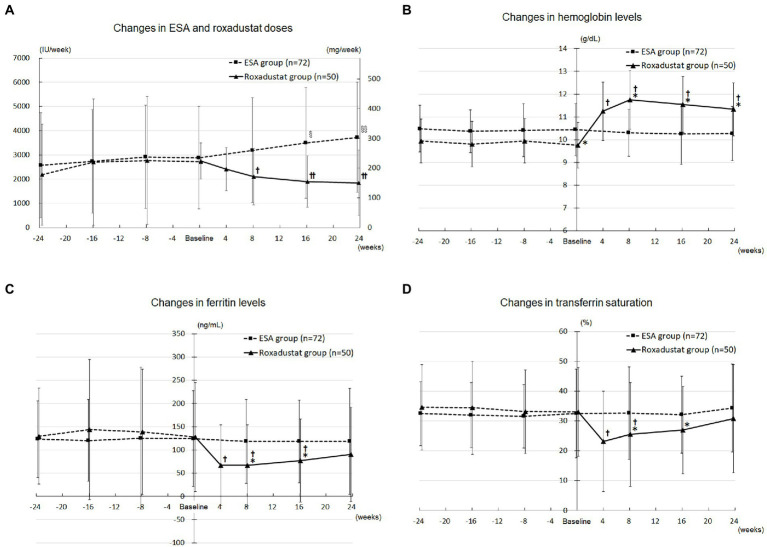
Anemia and iron metabolism. **(A)** Changes in the ESA (IU/wk) and roxadustat (mg/wk) doses administered during the study. **(B)** Changes in the hemoglobin levels in the roxadustat and ESA groups. **(C)** Changes in the ferritin levels in the roxadustat and ESA groups. **(D)** Changes in the transferrin saturation in the roxadustat and ESA groups. ESA, erythropoiesis-stimulating agent; IU, international units. §, *value of p* < 0.05 versus − 24 weeks, −16 weeks, and baseline; §§, *value of p* < 0.05 versus − 24 weeks, −16 weeks, −8 weeks, and baseline. †, *value of p* < 0.05 versus baseline; ††, *value of p* < 0.05 versus baseline and 4 weeks. *, *value of p* < 0.05 versus the ESA group.

Figure 3A depicts the changes in the doses of ESA and roxadustat in the two groups. In the ESA group, the ESA dose increased significantly from 2,882 ± 2,114 IU/wk. at baseline to 3,497 ± 2,280 IU/wk. at 16 weeks (*value of p* < 0.05) and 3,726 ± 2,273 IU/week. at 24 weeks (*value of p* < 0.05). In the roxadustat group, the roxadustat dose decreased significantly from 224 ± 60 mg/wk. at baseline to 171 ± 95 mg/wk. at 8 weeks (*value of p* < 0.05), 154 ± 86 mg/wk. at 16 weeks (*value of p* < 0.05), and 151 ± 110 mg/wk. at 24 weeks (*value of p* < 0.05).

### Roxadustat’s effect on anemia

The roxadustat-treated patients’ hemoglobin concentrations increased significantly from 9.8 ± 1.0 g/dL at baseline to 11.2 ± 1.3 g/dL at 4 weeks (*value of p* < 0.05), 11.7 ± 1.3 g/dL at 8 weeks (*value of p* < 0.05), 11.5 ± 1.2 g/dL at 16 weeks (*value of p* < 0.05), and 11.3 ± 1.2 g/dL at 24 weeks (*value of p* < 0.05), whereas the ESA group’s hemoglobin concentrations did not change significantly over the study term. As shown in Figure 3B, the roxadustat group’s baseline hemoglobin concentration was lower significantly than that of the ESA group (9.8 ± 1.0 g/dL versus 10.4 ± 1.0 g/dL, *value of p* < 0.05), but the roxadustat group’s value was higher significantly compared with the ESA group’s at 8 weeks (11.7 ± 1.3 g/dL versus 10.3 ± 1.0 g/dL, *value of p* < 0.05), 16 weeks (11.5 ± 1.2 g/dL versus 10.2 ± 0.9 g/dL, *value of p* < 0.05), and 24 weeks (11.3 ± 1.2 g/dL versus 10.3 ± 1.0 g/dL, *value of p* < 0.05).

### Factors associated with the change in hemoglobin levels during first 4 weeks of roxadustat administration

According to the simple linear regression analyses, the change in the patients’ hemoglobin levels during first 4 weeks of roxadustat administration was significantly correlated with statin use and the roxadustat dose at baseline. The multiple linear regression analysis using the variables that correlated significantly with the change in hemoglobin levels during first 4 weeks of roxadustat administration in the simple linear regression analyses (Table 2) revealed that only the roxadustat dose at baseline (standard coefficient [β] = 0.280, *value of p* = 0.043) was independently correlated with the change in hemoglobin levels during the first 4 weeks of roxadustat administration.

**Table 2:** Simple and multiple linear regression analyses of the variables correlated with the change in hemoglobin levels during the first 4 weeks of roxadustat administration.

Variables	Simple linear regression analysis		Multiple linear regression analysis	
	Standard coefficient	*p* value	Standard coefficient	*p* value
Age (years)	0.222	0.12		
Male sex (yes vs. no)	0.149	0.30		
Body mass index (kg/m^2^)	0.060	0.68		
Systolic blood pressure (mmHg)	0.011	0.94		
Diastolic blood pressure (mmHg)	0.010	0.94		
Diabetes mellitus (yes vs. no)	−0.159	0.27		
Hypertension (yes vs. no)	−0.010	0.95		
Previous myocardial infarction (yes vs. no)	0.031	0.83		
Previous stroke (yes vs. no)	0.030	0.83		
Calcium-containing phosphate binder use (yes vs. no)	−0.032	0.83		
Calcium-free phosphate binder use (yes vs. no)	−0.156	0.28		
Vitamin D analog use (yes vs. no)	−0.165	0.25		
Iron supplement use (yes vs. no)	0.159	0.27		
Zinc supplement use (yes vs. no)	0.194	0.18		
Statin use (yes vs. no)	0.290	0.041*	0.249	0.07
Ezetimibe use (yes vs. no)	0.200	0.16		
Estimated glomerular filtration rate at baseline (mL/min/1.73 m^2^)	−0.084	0.56		
Urinary protein excretion at baseline (g/gCr)	0.239	0.10		
Hemoglobin A1c at baseline (%)	−0.196	0.20		
Total cholesterol at baseline (mg/dL)	−0.053	0.72		
Low-density lipoprotein-cholesterol at baseline (mg/dL)	−0.037	0.80		
High-density lipoprotein-cholesterol at baseline (mg/dL)	−0.008	0.96		
Triglyceride at baseline (mg/dL)	−0.120	0.41		
Uric acid at baseline (mg/dL)	0.132	0.36		
Albumin at baseline (g/dL)	−0.171	0.24		
Hemoglobin at baseline (g/dL)	−0.226	0.11		
Sodium at baseline (mEq/L)	−0.108	0.46		
Potassium at baseline (mEq/L)	0.044	0.76		
Chloride at baseline (mEq/L)	0.150	0.30		
Total calcium at baseline (mg/dL)	−0.162	0.26		
Phosphorus at baseline (mg/dL)	−0.195	0.17		
Magnesium at baseline (mg/dL)	−0.261	0.07		
Brain natriuretic peptide at baseline (pg/mL)	−0.007	0.97		
C-reactive protein at baseline (mg/dL)	0.186	0.20		
Ferritin at baseline (ng/mL)	0.226	0.13		
Transferrin saturation at baseline (%)	0.014	0.93		
ESA (epoetin beta pegol vs. darbepoetin alfa)	−0.034	0.82		
ESA dose at baseline (IU/week)	0.079	0.58		
Erythropoietin resistance index at baseline (IU/week/kg/(g/dL)	0.105	0.47		
Roxadustat dose at baseline (mg/week)	0.316	0.025*	0.280	0.043*

ESA, erythropoiesis-stimulating agent; IU, international units. *Statistically significant.

### Factors associated with the hemoglobin concentration after 24 weeks of roxadustat administration

Simple linear regression analyses revealed that the hemoglobin concentration after 24 weeks of roxadustat administration was significantly correlated with age and the roxadustat dose at 24 weeks. With these two variables, we conducted a multiple linear regression analysis (Table 3), which revealed that age (β = 0.319, *value of p* = 0.017) and the roxadustat dose at 24 weeks (β = −0.347, *value of p* = 0.010) were each independently correlated with the hemoglobin concentration after 24 weeks of roxadustat administration.

**Table 3:** Simple and multiple linear regression analyses of the variables correlated with hemoglobin concentration after 24 weeks of roxadustat administration.

Variables	Simple linear regression analysis		Multiple linear regression analysis	
	Standard coefficient	*p* value	Standard coefficient	*p* value
Age (years)	0.308	0.030*	0.319	0.017*
Male sex (yes vs. no)	−0.112	0.44		
Body mass index (kg/m^2^)	0.041	0.78		
Systolic blood pressure (mmHg)	−0.069	0.64		
Diastolic blood pressure (mmHg)	−0.165	0.26		
Diabetes mellitus (yes vs. no)	0.060	0.68		
Hypertension (yes vs. no)	0.118	0.42		
Previous myocardial infarction (yes vs. no)	−0.012	0.93		
Previous stroke (yes vs. no)	0.011	0.94		
Calcium-containing phosphate binder use (yes vs. no)	−0.265	0.06		
Calcium-free phosphate binder use (yes vs. no)	0.177	0.22		
Vitamin D analog use (yes vs. no)	0.129	0.37		
Iron supplement use (yes vs. no)	0.136	0.35		
Zinc supplement use (yes vs. no)	0.010	0.94		
Statin use (yes vs. no)	0.208	0.15		
Ezetimibe use (yes vs. no)	−0.139	0.33		
Estimated glomerular filtration rate at baseline (mL/min/1.73 m^2^)	−0.005	0.97		
Urinary protein excretion at baseline (g/gCr)	−0.044	0.76		
Hemoglobin A1c at baseline (%)	0.204	0.18		
Total cholesterol at baseline (mg/dL)	−0.184	0.20		
Low-density lipoprotein-cholesterol at baseline (mg/dL)	−0.251	0.08		
High-density lipoprotein-cholesterol at baseline (mg/dL)	0.065	0.66		
Triglyceride at baseline (mg/dL)	−0.088	0.55		
Uric acid at baseline (mg/dL)	0.137	0.34		
Albumin at baseline (g/dL)	−0.025	0.86		
Hemoglobin at baseline (g/dL)	0.206	0.15		
Sodium at baseline (mEq/L)	−0.001	0.99		
Potassium at baseline (mEq/L)	−0.173	0.23		
Chloride at baseline (mEq/L)	−0.143	0.32		
Total calcium at baseline (mg/dL)	0.204	0.16		
Phosphorus at baseline (mg/dL)	−0.031	0.83		
Magnesium at baseline (mg/dL)	0.140	0.34		
Brain natriuretic peptide at baseline (pg/mL)	−0.251	0.17		
C-reactive protein at baseline (mg/dL)	0.121	0.41		
Ferritin at baseline (ng/mL)	0.120	0.42		
Transferrin saturation at baseline (%)	0.091	0.55		
ESA (epoetin beta pegol vs. darbepoetin alfa)	0.068	0.46		
ESA dose at baseline (IU/week)	−0.052	0.72		
Erythropoietin resistance index at baseline (IU/week/kg/(g/dL)	0.080	0.58		
Roxadustat dose at 24 weeks (mg/week)	−0.336	0.017*	−0.347	0.010*
Average dose of roxadustat during 24 weeks (mg/week)	−0.111	0.44		

ESA, erythropoiesis-stimulating agent; IU, international units. *Statistically significant.

### Roxadustat’s effect on iron metabolism

The roxadustat group’s ferritin concentration decreased significantly from 127.7 ± 117.3 ng/mL at baseline to 67.0 ± 86.5 ng/mL at 4 weeks (*value of p* < 0.05), 66.6 ± 87.6 ng/mL at 8 weeks (*value of p* < 0.05), and 76.9 ± 89.4 ng/mL at 16 weeks (*value of p* < 0.05), but it was not significantly different from baseline at 24 weeks. The ferritin concentrations in the ESA group at 8, 16, and 24 weeks were not significantly different from that at baseline. The roxadustat group’s ferritin concentration was significantly lower compared with the ESA group’s at 8 weeks (66.6 ± 87.6 ng/mL versus 118.4 ± 90.8 ng/mL, *value of p* < 0.05) and 16 weeks (76.9 ± 89.4 ng/mL versus 118.0 ± 99.7 ng/mL, *value of p* < 0.05; Figure 3C).

The roxadustat group’s TSAT decreased significantly from 33.0 ± 14.8 ng/mL at baseline to 23.1 ± 16.8 ng/mL at 4 weeks (*value of p* < 0.05) and 25.5 ± 17.4 ng/mL at 8 weeks (*value of p* < 0.05), but it did not significantly differ from baseline at 16 and 24 weeks. TSAT in the ESA group at 8, 16, and 24 weeks was not significantly different from that at baseline. The roxadustat group’s TSAT was significantly lower compared with the ESA group’s at 8 weeks (25.5 ± 17.4 ng/mL versus 32.6 ± 10.2 ng/mL, *value of p* < 0.05) and 16 weeks (26.9 ± 14.6 ng/mL versus 32.1 ± 12.4 ng/mL, *value of p* < 0.05; Figure 3D).

### Roxadustat’s effects on renal function and proteinuria

**Figure 4:**
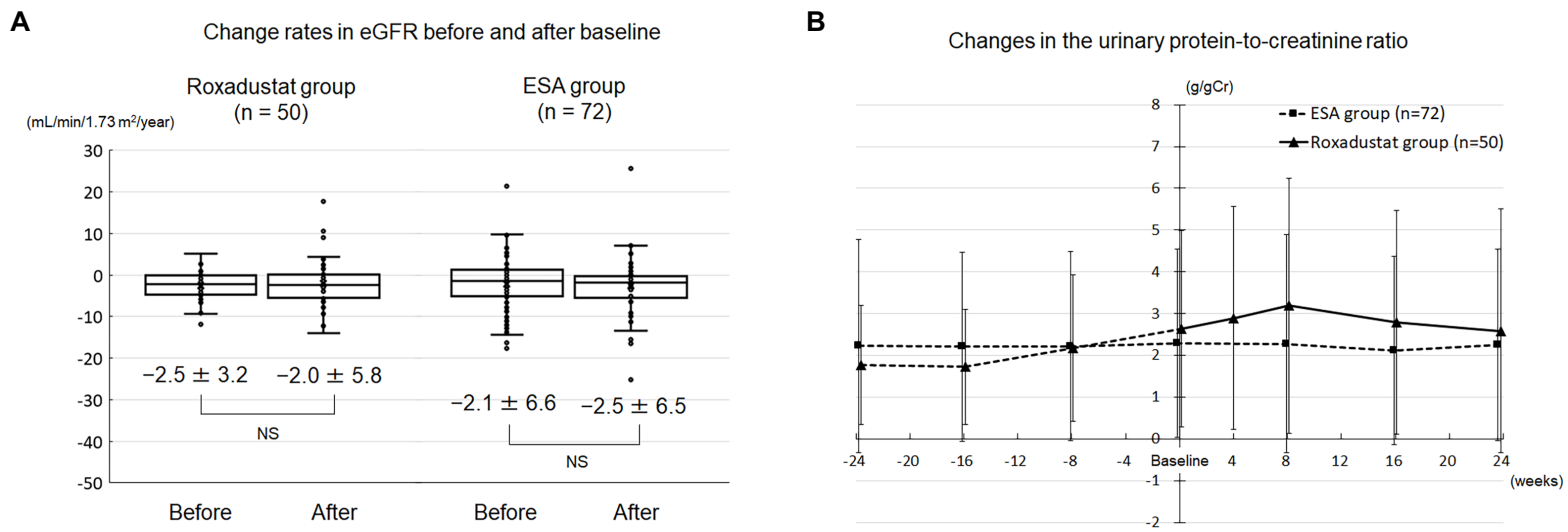
Renal function and proteinuria. **(A)** Change rates in the eGFR before and after baseline in the roxadustat and ESA groups. **(B)** Changes in the urinary protein-to-creatinine ratio in the roxadustat and ESA groups. eGFR, estimated glomerular filtration rate; ESA, erythropoiesis-stimulating agent; NS, not significant.

The change rate of eGFR did not differ significantly between before baseline and after baseline in both groups (Figure 4A). The urinary protein/creatinine ratio did not significantly change over the study term in either group (Figure 4B).

### Roxadustat’s effect on lipid metabolism

**Figure 5:**
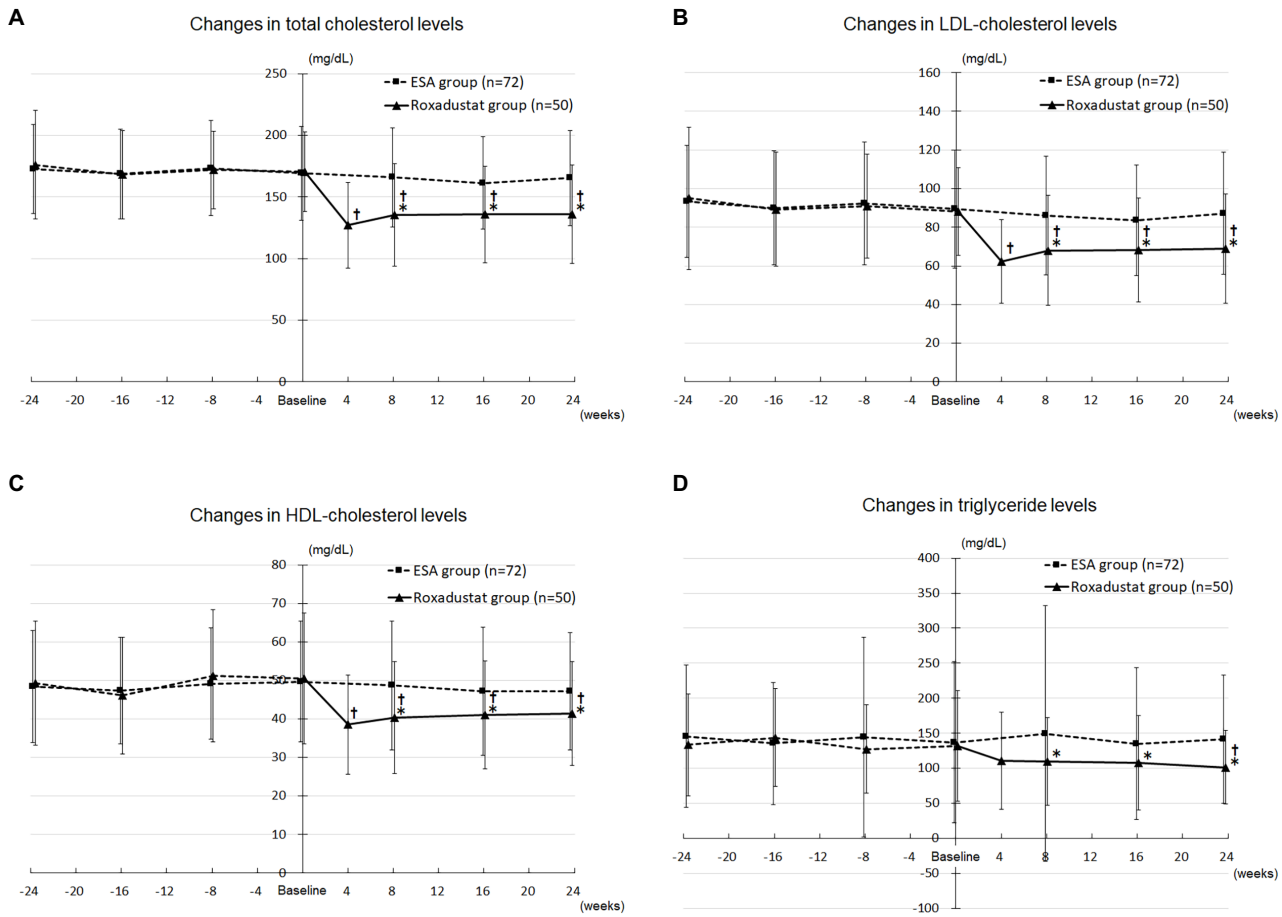
Lipid metabolism. **(A)** Changes in total cholesterol levels in the roxadustat and ESA groups. **(B)** Changes in LDL-cholesterol levels in the roxadustat and ESA groups. **(C)** Changes in HDL-cholesterol levels in the roxadustat and ESA groups. **(D)** Changes in triglyceride levels in the roxadustat and ESA groups. ESA, erythropoiesis-stimulating agent; HDL, high-density lipoprotein; LDL, low-density lipoprotein. *, *value of p* < 0.05 versus the ESA group; †, *value of p* < 0.05 versus baseline.

The roxadustat group’s TC concentration decreased significantly from 170.5 ± 32.4 mg/dL at baseline to 127.1 ± 34.9 mg/dL at 4 weeks (*value of p* < 0.05), 135.5 ± 41.7 mg/dL at 8 weeks (*value of p* < 0.05), 135.8 ± 39.3 mg/dL at 16 weeks (*value of p* < 0.05), and 135.9 ± 40.0 mg/dL at 24 weeks (*value of p* < 0.05), whereas the ESA group’s values did not change significantly over the study term. The roxadustat group’s TC concentration was significantly lower compared with the ESA group’s at 8 weeks (135.5 ± 41.7 mg/dL versus 165.9 ± 40.2 mg/dL, *value of p* < 0.05), 16 weeks (135.8 ± 39.3 mg/dL versus 161.3 ± 37.4 mg/dL, *value of p* < 0.05), and 24 weeks (135.9 ± 40.0 mg/dL versus 165.3 ± 38.4 mg/dL, *value of p* < 0.05; Figure 5A).

The roxadustat group’s LDL-C concentration decreased significantly from 88.1 ± 22.6 mg/dL at baseline to 62.3 ± 21.8 mg/dL at 4 weeks (*value of p* < 0.05), 67.9 ± 28.5 mg/dL at 8 weeks (*value of p* < 0.05), 68.3 ± 27.0 mg/dL at 16 weeks (*value of p* < 0.05), and 69.1 ± 28.3 mg/dL at 24 weeks (*value of p* < 0.05), whereas the ESA group’s values did not change significantly over the study term. The roxadustat group’s LDL-C concentration was significantly lower compared with the ESA group’s at 8 weeks (67.9 ± 28.5 mg/dL versus 86.0 ± 30.7 mg/dL, *value of p* < 0.05), 16 weeks (68.3 ± 27.0 mg/dL versus 83.6 ± 28.5 mg/dL, *value of p* < 0.05), and 24 weeks (69.1 ± 28.3 mg/dL versus 87.2 ± 31.5 mg/dL, *value of p* < 0.05; Figure 5B).

The roxadustat group’s HDL-C concentration decreased significantly from 50.5 ± 17.0 mg/dL at baseline to 38.5 ± 13.0 mg/dL at 4 weeks (*value of p* < 0.05), 40.3 ± 14.5 mg/dL at 8 weeks (*value of p* < 0.05), 41.1 ± 14.0 mg/dL at 16 weeks (*value of p* < 0.05), and 41.4 ± 13.5 mg/dL at 24 weeks (*value of p* < 0.05), whereas the ESA group’s values did not change significantly over the study term. The roxadustat group’s HDL-C concentration was significantly lower compared with the ESA group’s at 8 weeks (40.3 ± 14.5 mg/dL versus 48.7 ± 16.8 mg/dL, *value of p* < 0.05), 16 weeks (41.1 ± 14.0 mg/dL versus 47.2 ± 16.7 mg/dL, *value of p* < 0.05), and 24 weeks (41.4 ± 13.5 mg/dL versus 47.2 ± 15.3 mg/dL, *value of p* < 0.05; Figure 5C).

The roxadustat group’s triglyceride concentration decreased significantly from 132.2 ± 78.9 mg/dL at baseline to 101.5 ± 52.7 mg/dL at 24 weeks (*value of p* < 0.05), whereas the ESA group’s values did not change significantly over the study term. The roxadustat group’s triglyceride concentration was significantly lower compared with the ESA group’s at 8 weeks (109.6 ± 62.6 mg/dL versus 149.6 ± 182.7 mg/dL, *value of p* < 0.05), 16 weeks (108.0 ± 67.1 mg/dL versus 135.1 ± 108.2 mg/dL, *value of p* < 0.05), and 24 weeks (101.5 ± 52.7 mg/dL versus 141.6 ± 91.4 mg/dL, *value of p* < 0.05; Figure 5D).

### Changes of other clinical parameters and clinical adverse effects

We detected no significant changes over the study term in the patients’ body mass, systolic and diastolic blood pressure value, albumin, hemoglobin A1c, uric acid, sodium, potassium, chloride, total calcium, phosphorus, magnesium, brain natriuretic peptide, or C-reactive protein (data not shown). Ten of the patients in the roxadustat group experienced an adverse effect: nausea, *n* = 3; dizziness, *n* = 2; deep vein thrombosis, *n* = 2; diarrhea, *n* = 1; anorexia, *n* = 1; hepatotoxicity, *n* = 1. The roxadustat treatment of these patients was thus discontinued. None of the patients in the ESA group experienced any adverse effects during the study period.

## Discussion

The results of the present study demonstrated that roxadustat improved anemia and reduced serum cholesterol and triglyceride levels in patients who were not undergoing renal replacement therapy after changing from ESA treatment. Our analyses also revealed that the roxadustat dose at baseline was correlated with the change in hemoglobin levels during the first 4 weeks of roxadustat administration, whereas age and the roxadustat dose at 24 weeks were correlated with the hemoglobin concentration after 24 weeks of roxadustat administration. However, roxadustat did not improve iron metabolism or affect renal function or proteinuria in these patients.

Roxadustat is a HIF prolyl hydroxylase inhibitor which stabilizes HIF and stimulates the expression of erythropoiesis-related genes. It increases the erythropoietin concentration within physiologic range in the liver and kidneys, thereby increasing or maintaining the hemoglobin concentration in CKD patients with anemia (5). A recent randomized clinical trial revealed that roxadustat sustains the hemoglobin concentration in patients with non-dialysis CKD who were switched from an ESA (7). In the present study, roxadustat increased and stabilized the hemoglobin concentration after the patients’ treatment was switched from an ESA in a similar patient group. The required dose of ESA continued to increase in the ESA group during the study period but the dose of roxadustat did not require to be increased in the roxadustat group after patients were switched from their ESA. These findings indicate that roxadustat improves and helps stabilize hemoglobin concentrations in patients with non-dialysis CKD plus anemia who were receiving an ESA on a clinical practice. In the present study, only the initial roxadustat dose was associated with the change in hemoglobin levels during the first 4 weeks of roxadustat administration. A phase 2 clinical study reported that roxadustat increased hemoglobin concentrations in a dose-dependent manner in patients with non-dialysis CKD (11). These findings indicate that roxadustat dose-dependently improves anemia in patients with non-dialysis CKD regardless of patient demographic and clinical characteristics. In the present study, age and the roxadustat dose at 24 weeks were associated with the hemoglobin concentration after 24 weeks of roxadustat administration. It has been demonstrated that age was correlated positively with the hemoglobin concentration in patients on hemodialysis receiving ESA (12). In our study, age was positively correlated with the hemoglobin concentration in patients with non-dialysis CKD who were taking roxadustat. These results suggest that both ESA and roxadustat might be more effective in older patients than in younger patients. Several study reported that the immunohistochemical expression of erythropoietin receptor was higher in older patients than in younger patients (13, 14). This might explain the findings that ESA and roxadustat were more effective in older patients than in younger patients. It has been reported that the dose of ESA was correlated negatively with the hemoglobin concentration in patients on hemodialysis (15). In our study, the roxadustat dose was negatively correlated with the hemoglobin concentration in patients with non-dialysis CKD. These negative correlations between the doses of ESA and roxadustat and the hemoglobin concentration might be because patients with severe anemia were treated with higher doses of ESA or roxadustat. Further research is necessary to elucidate the relationship between the roxadustat dose and hemoglobin concentration in patients with non-dialysis CKD.

Roxadustat was indicated to improve patient’s iron metabolism *via* multi-pathways. It increases ion absorption from the gastrointestinal tract, enhances release of iron from the hepatocytes, and increases serum total binding capacity of iron (5). A recent phase 3 clinical study reported that roxadustat reduced ferritin levels in serum and TSAT in patients with non-dialysis CKD (16). However, in the present study, we detected no significant differences in ferritin levels and TSAT between the roxadustat and ESA groups, although ferritin levels and TSAT at 8 and 16 weeks were lower significantly in the roxadustat group than in the ESA group. Several reasons can be considered to explain such differences between our present findings and those of earlier studies. First, the roxadustat dose was reduced after treatment initiation because anemia was improved, which might have contributed to the increases of ferritin levels and TSAT in the late phase of the study term. Second, iron supplementation was started in 10 patients after roxadustat initiation because of the improvement of iron metabolism, which might have been responsible for the increases of ferritin levels and TSAT in the late phase of the study term. Further research is necessary to elucidate roxadustat’s effect on iron metabolism in patients with non-dialysis CKD who switched from an ESA on a clinical practice.

Roxadustat was demonstrated to reduce serum cholesterol levels in a dose-dependent manner regardless of baseline statin use (17). A recent phase 3 clinical study reported that roxadustat reduced serum TC, LDL-C, HDL-C, and triglyceride concentrations in patients on hemodialysis (8). In the present study, roxadustat reduced serum TC, LDL-C, HDL-C, and triglyceride concentrations in patients with non-dialysis CKD. These results may indicate that roxadustat reduces serum cholesterol and triglyceride levels in both dialysis-dependent and dialysis-independent patients with CKD. HIF-1 accelerates degradation of HMG-CoA reductase in the liver through activation of insulin-induced gene 2 transcription, leading to reduced cholesterol synthesis (18). HIF-2 inhibits ATP-binding cassette transporter A1 gene expression, which attenuates the formation of HDL-C (19). HIF-1 also stimulates lipin 1 gene expression, which contributes to triglyceride accumulation in cells (20). Therefore, roxadustat stabilizes HIF and then may reduce TC, LDL-C, HDL-C, and triglyceride levels by these mechanisms.

Roxadustat has been reported to have several clinical side effects including digestive tract disorders, dizziness, and thrombosis (21, 22). Among the present study’s patients treated with roxadustat, five patients had gastrointestinal symptoms, two patients reported dizziness, and two patients developed deep vein thrombosis. Phase 2 and 3 clinical trials involving non-dialysis CKD patients showed that the rates of digestive tract symptoms, dizziness, and deep vein thrombosis were 29.7, 6.2, and 1.2%, respectively (21, 22), which are compatible with our study findings. We also observed that roxadustat did not influence the renal function or the proteinuria of patients with non-dialysis CKD. These results indicate that roxadustat can be administered safely in patients with non-dialysis CKD.

The present study had several advantages over the previously reported study (7). First, we evaluated roxadustat’s effects on renal function, proteinuria, and lipid metabolism. Second, we also identified the associated factors of the change in hemoglobin levels after roxadustat treatment in patients with non-dialysis CKD who were switched from an ESA. Therefore, our findings may be valuable for further research on the application of roxadustat in patients with non-dialysis CKD.

The present study was different from our previous study in two aspects (23). First, in our previous study, we assessed roxadustat’s effects on iron metabolism, anemia, residual renal function, and peritoneal membrane function in peritoneal dialysis patients. By contrast, in the present study, roxadustat’s effects on anemia, iron metabolism, renal function, proteinuria, and lipid metabolism were investigated in non-dialysis CKD patients. Therefore, the study population was totally different between our previous and present studies. Second, in our previous study, we assessed the factors that might be associated with the roxadustat dose in peritoneal dialysis patients. By contrast, in the present study, we assessed the factors that might be associated with the change in hemoglobin levels after roxadustat administration in non-dialysis CKD patients. Therefore, the clinical outcome was fundamentally different between our previous and present studies. Therefore, our present findings could be used in future studies regarding roxadustat’s effects on anemia, iron metabolism, renal function, proteinuria, and lipid metabolism and the factors that may be associated with the improvement in anemia in patients with non-dialysis CKD.

Several study limitations should be addressed. First, this study was a retrospective and observational study; therefore, sample selection bias could not have been completely avoided. Second, the patients were recruited from a single institution, which limits the results’ external validness. Third, the number of patients (*n* = 122) was small, and this decreases the statistical power for detecting between-group differences. Fourth, in Japan, roxadustat prescription is not restricted by any medical insurances if there are approved indications. In this study, an ESA was switched to roxadustat based on physician’s judgment or patient’s preference. However, there were significant differences in baseline characteristics including percentage of diabetes mellitus and hemoglobin concentration between the two groups. The lack of similarity regarding patients’ characteristics between the two groups might have affected the study results. Fifth, the long-term effects of roxadustat on renal function, proteinuria, and lipid metabolism were not examined. Large, long-term, prospective, randomized studies are necessary to establish roxadustat’s effects on renal function, proteinuria, and lipid metabolism and to decide which factors are associated with the change in hemoglobin levels after roxadustat administration in patients with non-dialysis CKD.

In conclusion, roxadustat improved anemia and reduced serum cholesterol and triglyceride levels in patients who were not undergoing renal replacement therapy after switching from an ESA without affecting renal function or proteinuria. The results obtained in this study indicate that roxadustat might be superior to ESAs regarding improvement effects on anemia and lipid metabolism in patients with non-dialysis CKD.

## Data availability statement

The raw data supporting the conclusions of this article will be made available by the authors, without undue reservation.

## Ethics statement

The studies involving human participants were reviewed and approved by Ethics Committee of Saitama Medical Center, Jichi Medical University. Written informed consent for participation was not required for this study in accordance with the national legislation and the institutional requirements.

## Author contributions

KH and SK conceived of the study and designed the study. SM, KY, MH, and TK collected the data. KI and YU performed the statistical analysis. KH wrote the manuscript’s first draft. SO conducted critical revisions. YM endorsed the manuscript’s final version. All authors contributed to the study and manuscript and approved the manuscript’s final version.

## Conflict of interest

The authors declare that the research was conducted in the absence of any commercial or financial relationships that could be construed as a potential conflict of interest.

## Publisher’s note

All claims expressed in this article are solely those of the authors and do not necessarily represent those of their affiliated organizations, or those of the publisher, the editors and the reviewers. Any product that may be evaluated in this article, or claim that may be made by its manufacturer, is not guaranteed or endorsed by the publisher.
